# Synthesis of an acridine orange sulfonamide derivative with potent carbonic anhydrase IX inhibitory action

**DOI:** 10.1080/14756366.2017.1302441

**Published:** 2017-03-23

**Authors:** Marco Bragagni, Fabrizio Carta, Sameh M. Osman, Zeid AlOthman, Claudiu T. Supuran

**Affiliations:** a Dipartimento Neurofarba, Sezione di Scienze Farmaceutiche e Nutraceutiche, Università degli Studi di Firenze, Sesto Fiorentino, FlorenceItaly;; b Department of Chemistry, College of Science, King Saud University, Riyadh, Saudi Arabia

**Keywords:** Carbonic anhydrase, sulfonamide, inhibitor, tumor, acridine orange

## Abstract

Acridine orange (AO) a fluorescent cationic dye used for the management of human musculoskeletal sarcomas, due to its strong tumoricidal action and accumulation in the acidic environment typical of hypoxic tumors, was used for the preparation of a primary sulfonamide derivative. The rationale behind the drug design is the fact that hypoxic, acidic tumors overexpress carbonic anhydrase (CA, EC 4.2.1.1) isoforms, such as CA IX, which is involved in pH regulation, proliferation, cell migration and invasion, and this enzyme is strongly inhibited by primary sulfonamides. The AO-sulfonamide derivative was indeed a potent, low nanomolar CA IX inhibitor whereas its inhibition of the cytosolic isoforms CA I and II was in the micromolar range. A second transmembrane, tumor-associated isoform, CA XII, was also effectively inhibited by the AO-sulfonamide derivative, making this compound an interesting theranostic agent for the management of hypoxic tumors.

## Introduction

Acridine orange (**AO**) is a heterocyclic derivative used as a nucleic acid-selective fluorescent cationic dye useful for cell cycle determination, as it interacts with DNA and RNA by intercalation within the double helix or by electrostatic attractions to the negatively charged phosphate groups, respectively. It also enters acidic compartments such as lysosomes, becoming protonated and sequestered inside that region of the cell/tissue. In such low pH conditions, the dye emits orange light when excited by blue light, being used to identify engulfed apoptotic cells^1–3^.
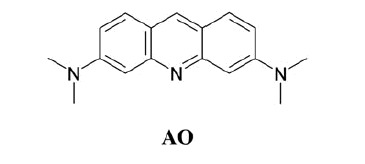


In the last years, Kusuzaki’s and Baldini’s groups found that AO accumulates in the musculoskeletal sarcomas^1–6^. After illumination of the tumors loaded with **AO** with visible light (or irradiation with low-dose X-rays), the dye rapidly exerted a selective killing of the cancer cells^1–6^. Thus, **AO** in combination with surgery and photodynamic (PD) or radiodynamic (RD) therapies has been proposed as an alternative approach for the management of human musculoskeletal sarcomas, due to its strong tumoricidal action following excitation with a light source at 466 nm, with promising results being obtained mainly in Japan^1–6^.

Osteosarcomas^7^ as many other tumor types were shown to overexpress the metalloenzyme carbonic anhydrase (CA, EC 4.2.1.1)^8–15^ involved in several processes related to tumorigenesis, tumor progression and metastases formation^8^^,^^9^. CAs are highly effective catalysts for the hydration of carbon dioxide with the formation of bicarbonate and protons, being widespread in all life kingdoms, with seven genetically distinct families known to date^15–21^. By catalyzing the reversible CO_2_ hydration to bicarbonate and protons, the CAs are involved in many physiological processes connected with electrolyte secretion^22–24^, pH regulation^25–29^, tumorigenesis^30–33^, etc., and their inhibition leads to pharmacological effects^34–40^. Indeed, sulfonamide CA inhibitors (CAIs) are clinically used as diuretics, antiglaucoma, anticonvulsant, antiobesity and antitumor agents^34–44^. Many drug design strategies are presently available for designing effective and isoform-selective such agents^45–50^, but the primary sulfonamides remain among the most investigated CAIs due to their high affinity for many CA isoforms of pharmacologic interest, rather convenient pharmacology and ease of preparation^45–50^.

Here, we report a study in which we designed a compound which might combine the affinity of AO for the tumors and the fact that many of them overexpress CA isoforms involved in tumorigenesis (e.g. CA IX and XII)^9^^,^^19^. The designed compound incorporates both **AO** and sulfonamide moieties, which have affinity for the CAs. The **AO**-sulfonamide agent reported here could represent a theranostic agent for the management of hypoxic tumors.

## Material and methods

### Chemistry

Anhydrous solvents and all reagents were purchased from Sigma-Aldrich (Milan, Italy). All reactions involving air- or moisture-sensitive compounds were performed under a nitrogen atmosphere using dried glassware and syringes techniques to transfer solutions. Nuclear magnetic resonance (^1^H NMR, ^13^C NMR) spectra were recorded using a Bruker Advance III 400 MHz spectrometer in DMSO-d_6_. Chemical shifts are reported in parts per million (ppm) and the coupling constants (*J*) are expressed in Hertz (Hz). Splitting patterns are designated as follows: s, singlet; d, doublet; triplet; q, quadruplet; dd, double of doublet. The assignment of exchangeable protons (O*H* and N*H*) was confirmed by the addition of D_2_O.

Synthesis of 3,6-bis(dimethylamino)-9-acridanthione **1**^51^
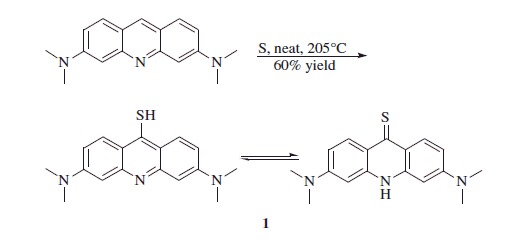


Acridine orange (3.0 g, 1.0 eq) and sulfur (0.43 g, 1.2 eq) were accurately mixed in a mortar and transferred to a pear shaped flask, which was preheated at 205 °C in a sand bath. The flask was maintained open at this temperature for 30 min. The dark purple residue formed was cooled down to r.t. and re-crystallized from DMF to afford the title compound **1** as a dark brown solid.

3,6-Bis(dimethylamino)-9-acridanthione **1**: 60% yield; ^1^H NMR: *δ*_H_ (400 MHz, DMSO-d_6_) 3.11 (s, 12H), 6.42 (s, 2H), 6.89 (d, *J* = 9.6 Hz, 2H), 8.67 (d, *J* = 9.6 Hz, 2H), 11.68 (s, 1H). ^13^C NMR: *δ*_C_ (100 MHz, DMSO-*d_6_*) 40.5, 94.2, 111.0, 120.9, 132.1, 139.1, 153.6. *m/z* (ESI positive) 298.13 [M + H]^+^.

Synthesis of 3,6-bis(dimethylamino)-9-(methylthio)acridine **2**^52^
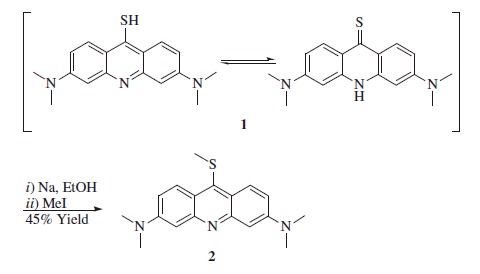


Sodium (0.38 g, 1.3 eq) was dissolved at r.t. in anhydrous ethanol (20 ml) under a nitrogen atmosphere, followed by the addition of 3,6-bis(dimethylamino)-9-acridanthione **1** (2 g, 1.0 eq). The reaction mixture was stirred under reflux for 30 min, cooled down to 50 °C and methyl iodide (1.15 g, 1.2 eq) was added drop-wise. The reaction mixture was left stirring o.n. at r.t. and filtrated. The filtrated solution was boiled, treated with decolorizing charcoal, filtered and heated again to boiling. Hot distilled water (5 ml) was added drop-wise under stirring. The solution was cooled down and maintained at 4 °C o.n. The red crystals which precipitated were recuperated by filtration and dried under *vacuo*.

3,6-Bis(dimethylamino)-9-(methylthio)acridine **2**: 45% yield; ^1^H NMR: *δ*_H_ (400 MHz, DMSO-d_6_) 2.48 (s, 3H), 3.13 (s, 12H), 6.90 (d, *J* = 2.8 Hz, 2H), 7.37 (dd, *J* = 9.6 2.8 Hz, 2H), 8.41 (d, *J* = 9.6 Hz, 2H). ^13^C NMR: *δ*_C_ (100 MHz, DMSO-d_6_) 21.3, 41.1, 104.2, 117.8, 120.9, 128.4, 143.3, 151.9, 152.5. *m/z* (ESI positive) 312.15 [M + H]^+^.

Synthesis of 4-(2-((3,6-bis(dimethylamino)acridin-9-yl)amino)ethyl)benzenesulfonamide perchlorate **3**.
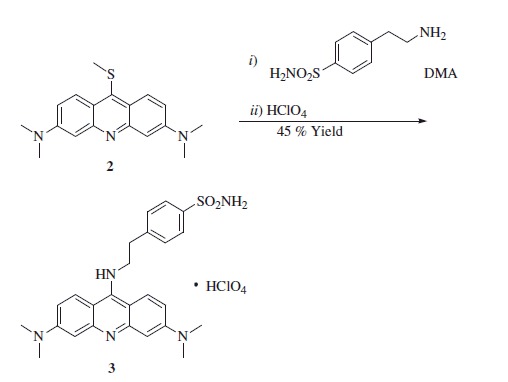


3,6-Bis(dimethylamino)-9-(methylthio)acridine **2** (0.50 g, 1.0 eq) and 4-(2-aminoethyl)benzenesulfonamide (0.96 g, 3 eq) were dissolved in anhydrous DMA (20 ml) at 140 °C. The reaction mixture was stirred at the same temperature for 2 h under a nitrogen atmosphere, cooled down to r.t. and a 6 M aqueous solution of perchloric acid (0.48 g, 3.0 eq) was added. The mixture was maintained at 0 °C for 1 h and the dark red crystals obtained were collected by centrifugation, washed with water and dried under *vacuo*.

4-(2-((3,6-Bis(dimethylamino)acridin-9-yl)amino)ethyl)benzenesulfonamide perchlorate **3**: 45% yield; ^1^H NMR: *δ*_H_ (400 MHz, DMSO-d_6_) 3.16 (s, 12H), 3.25 (t, *J* = 7.6 Hz, 2H), 4.21 (q, *J* = 7.6 Hz, 2H), 6.52 (d, *J* = 2.8 Hz, 2H), 7.02 (dd, *J* = 9.6 2.8 Hz, 2H), 7.34 (s, 2H), 7.50 (d, *J* = 8.4 Hz, 2H), 7.75 (d, *J* = 8.4 Hz, 2H), 8.22 (d, *J* = 9.6 Hz, 2H), 8.46 (t, 1H), 12.2 (s, 1H). ^13^C NMR: *δ*_C_ (100 MHz, DMSO-d_6_) 36.1, 40.3, 50.5, 95.0, 104.5, 112.1, 126.8, 128.0, 130.3, 143.2, 143.5, 143.6, 154.3, 155.2. *m/z* (ESI positive) 464.20 [M − ClO_4_]^+^.

Synthesis of *N*,N*,N',N'*-tetramethyl-*N''*-phenethyl-acridine-3,6,9-triamine **4**.
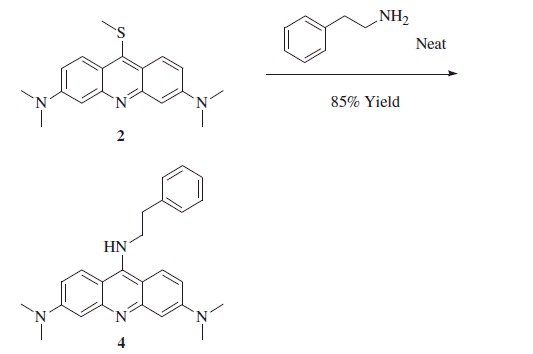


3,6-Bis(dimethylamino)-9-(methylthio)acridine **2** (0.4 g, 1.0 eq) was introduced in a pear shaped flask followed by phenylethylamine (0.47 g, 3.0 eq) at r.t. Then the flask was transferred to a sand bath, pre-heated at 140 °C, and left open at this temperature for 1.5 h. The dark residue was cooled-down to r.t., crushed with a spatula, repeatedly washed with water and dried under *vacuo*.

*N,N,N',N'*-tetramethyl-*N''*-phenethyl-acridine-3,6,9-triamine **4**: 85% yield; ^1^H NMR: *δ*_H_ (400 MHz, DMSO-d_6_) 3.04 (t, *J* = 7.6 Hz), 3.06 (s, 12H), 3.93 (q, *J* = 7.6 Hz, 2H), 6.64 (d, 2H), 6.92 (dd, *J* = 9.6 2.8 Hz, 2H), 7.29 (m, 5H), 8.05 (d, *J* = 9.6 Hz, 2H). ^13^C NMR: *δ*_C_ (100 MHz, DMSO-d_6_) 36.4, 40.5, 51.0, 95.2, 104.6, 112.0, 127.6, 127.9, 129.6, 129.8, 139.4, 143.4, 154.2, 155.1. *m/z* (ESI positive) 385.23 [M + H]^+^.

### Carbonic anhydrase assay

A stopped-flow method^53^ has been used for assaying the CA catalyzed CO_2_ hydration activity with Phenol red as indicator, working at the absorbance maximum of 557 nm, following the initial rates of the CA-catalyzed CO_2_ hydration reaction for 10–100 s. For each inhibitor at least six traces of the initial 5–10% of the reaction have been used for determining the initial velocity. The uncatalyzed rates were determined in the same manner and subtracted from the total observed rates. Stock solutions of inhibitor (0.01 mM) were prepared in distilled-deionized water with 5% DMSO and dilutions up to 0.1 nM were done thereafter with the assay buffer. The Inhibition constant (*K*_I_) was obtained by considering the classical Michaelis–Menten equation which has been fitted by non-linear least squares by using PRISM 3. All CA isozymes used in the experiments were purified recombinant proteins obtained as reported earlier by our group^54–64^.

## Results and discussion

The rationale of this work was to design a hybrid molecule which may show enhanced affinity for tumor cells due to the presence of both **AO** and sulfonamide moieties in its molecule. In addition, these hybrid compounds may retain the fluorescent properties of **AO**, and thus could be useful for PD and/or RD therapies, but these aspects are not investigated in this paper.

**Scheme 1.**
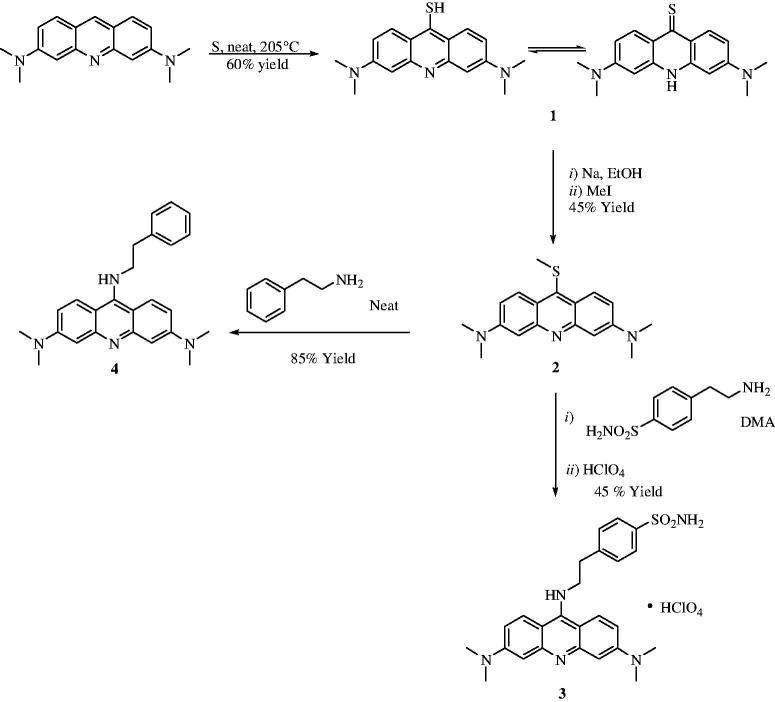
General synthetic procedure for 4-(2-((3,6-bis(dimethylamino)acridin-9-yl)amino)ethyl)benzenesulfonamide perchlorate **3** and *N,N,N',N'*-tetramethyl-*N''*-phenethyl-acridine-3,6,9-triamine **4**.

The synthetic procedure for obtaining the hybrid is shown in Scheme 1. Acridine was reacted with elemental sulfur, leading to the thiol/thione derivative **1**, which was methylated at the sulfur atom with methyl iodide, leading to the key methylthio-intermediate **2**^51^^,^^52^.

Reaction of **2** with amines, such as phenethylamine or 4-aminoethylbenzensulfonamide, led to the heterocyclic amines **3** and **4**, one incorporating the primary sulfonamide moiety (compound **3**) and the other one possessing exactly the same scaffold as **3**, but without the sulfonamide group (compound **4**), Scheme 1. Derivative **4** is in fact useful as a negative control in the enzyme inhibition experiments reported here (see later in the paper). Since all the purification procedures used to isolate the derivative **3** as the free base were unsuccessful, we converted the *in situ* formed free-base **3** (TLC monitoring) to its corresponding perchlorate salt, which precipitated at 0 °C within 1 h to afford the desired compound in good yield and excellent purity. The use of perchlorate salts for the isolation as well as purification of small molecule compounds as CAIs is well reported, also in consideration that the inorganic counterion does not have any effect on the CAs activity^7–9^.

### Carbonic anhydrase inhibition

We assessed the CA inhibitory activity of compounds **3** and **4**, using the clinically used drug acetazolamide (5-acetamido-1,3,4-thiadiazole-2-sulfonamide, **AAZ**) as positive control, for the inhibition of four human (h) isoforms, hCA I and II (cytosolic, widely distributed enzymes) as well as hCA IX and XII (transmembrane, tumor-associated enzymes) – Table 1.

**Table 1.** hCA I, II; IX and XII inhibition data with compounds **3**, **4**, **AAZ** and **AO**, by a stopped-flow CO_2_ hydrase assay^53^.

	*K*_I_ (nM)^a^			
Compound	hCA I	hCA II	hCA IX	hCA XII
**3**	7680	1650	9.1	4.9
**4**	>50,000	>50,000	>50,000	>50,000
**AAZ**	250	12	25.1	5.6
**AO**	>50,000	>50,000	>50,000	>50,000

a Errors in the range of 5% of the reported values, from three different determinations (data not shown).

Data of Table 1 show that **AO** and its non-sulfonamide derivative **4**, did not inhibit any CA isoform investigated here, whereas sulfonamides **3** and **AAZ** acted as inhibitors. The **AO**-sulfonamide hybrid **3** was a micromolar inhibitor of the cytosolic isoforms hCA I and II, with inhibition constants of 1.65–7.68 µM, whereas the transmembrane, tumor-associated isoforms hCA IX and XII were much more effectively inhibited, with inhibition constants of 4.9–9.1 nM. Acetazolamide was a medium potency hCA I inhibitor and a highly effective one for the remaining three isoforms, with inhibition constants of 5.6–25.1 nM (Table 1). These data show that the **AO**-sulfonamide hybrid **3** is a tumor-associated CA isoforms selective inhibitor, making it a valuable candidate for theranostic applications in the field of hypoxic tumors.

## Conclusions

We report here the synthesis and enzyme inhibition data of acridine orange, a fluorescent cationic dye used for the management of human musculoskeletal sarcomas, as well as those of a new compound based on the **AO** scaffold on which a sulfonamide zinc-binding moiety was introduced by using an original procedure. Due to the strong tumoricidal action and accumulation in the acidic environment (typical of hypoxic tumors) of **AO**, we designed the hybrid in such a way as to incorporate an additional functionality which may lead to interaction with hypoxic tumors, many of which overexpress CA IX and XII. Such enzymes are in fact involved in pH regulation, proliferation, cell migration and invasion in many cancer types. The reported **AO**-sulfonamide derivative was indeed a potent, low nanomolar CA IX inhibitor whereas its inhibition of the cytosolic isoforms CA I and II was in the micromolar range. A second transmembrane, tumor-associated isoform, CA XII, was also effectively inhibited by the **AO**-sulfonamide derivative, making this compound an interesting theranostic agent for the management of hypoxic tumors.
